# Enabling Nurse-Patient Communication With a Mobile App: Controlled Pretest-Posttest Study With Nurses and Non–English-Speaking Patients

**DOI:** 10.2196/19709

**Published:** 2021-07-30

**Authors:** David Silvera-Tawil, Courtney Pocock, DanaKai Bradford, Andrea Donnell, Jill Freyne, Karen Harrap, Sally Brinkmann

**Affiliations:** 1 Australian e-Health Research Centre Commonwealth Scientific and Industrial Research Organisation Marsfield Australia; 2 Western Health Melbourne Australia; 3 Australian e-Health Research Centre Commonwealth Scientific and Industrial Research Organisation Pullenvale Australia; 4 Australian e-Health Research Centre Commonwealth Scientific and Industrial Research Organisation Herston Australia

**Keywords:** nursing, interpersonal communication, mobile app, information technology, communication barrier, diversity, interpreters, mHealth, mobile phone

## Abstract

**Background:**

There is growing concern regarding the implications of miscommunication in health care settings, the results of which can have serious detrimental impacts on patient safety and health outcomes. Effective communication between nurses and patients is integral in the delivery of timely, competent, and safe care. In a hospital environment where care is delivered 24 hours a day, interpreters are not always available. In 2014, we developed a communication app to support patients’ interactions with allied health clinicians when interpreters are not present. In 2017, we expanded this app to meet the needs of the nursing workforce. The app contains a fixed set of phrases translated into common languages, and communication is supported by text, images, audio content, and video content.

**Objective:**

This study aims to evaluate the efficacy of the communication app to support nursing staff during the provision of standard care to patients from non–English-speaking backgrounds when an interpreter is not available.

**Methods:**

This study used a one-group pretest-posttest sequential explanatory mixed methods research design, with quantitative data analyzed using inferential statistics and qualitative data analyzed via thematic content analysis. A total of 134 observation sessions (82 pretest and 52 posttest) of everyday nurse-patient interactions and 396 app use sessions were recorded. In addition, a total of 134 surveys (82 pretest and 52 posttest) with nursing staff, 7 interviews with patients, and 3 focus groups with a total of 9 nursing staff participants were held between January and November 2017.

**Results:**

In the absence of the app, baseline interactions with patients from English-speaking backgrounds were rated as more successful (t_80_=5.69; *P*<.001) than interactions with patients from non–English-speaking backgrounds. When staff used the app during the live trial, interactions with patients from non–English-speaking backgrounds were rated as more successful than interactions without the app (*F*_2,119_=8.17; *P*<.001; η^2^=0.37). In addition, the level of staff frustration was rated lower when the app was used to communicate (t_80_=2.71; *P*=.008; *r*=0.29). Most participants indicated that the app assisted them in communicating.

**Conclusions:**

Through the use of the app, a number of patients from non–English-speaking backgrounds experienced better provision of standard care, similar to their English-speaking peers. Thus, the app can be seen as contributing to the delivery of equitable health care.

## Introduction

### Background

Good communication in clinical settings affects a number of outcomes, such as patient satisfaction and adherence and, consequently, health outcomes [1]. One of the challenging areas of health care communication is communication with culturally and linguistically diverse (CALD) patients [1,2]. In acute hospital settings, suboptimal communication appears to be the largest source of preventable medical errors [3].

In hospital settings, delays for CALD patients are common, as clinicians require interpreter services, and for a variety of reasons, this may not be immediately available [3]. Using ad hoc translators, such as family members or friends, can impact the quality of care and confidentiality, and increase distress and conflict [3]. The demand for professional interpreter services can exceed supply due to the limited number of qualified interpreters and the increasing number of patients from non–English-speaking backgrounds (NESBs). When clinical staff are unable to communicate directly with patients, their ability to adequately respond to patient needs has the potential to impact patient care and experience [4-6]. There is also a risk to patients’ mental health as a result of loneliness and isolation due to their inability to communicate with either staff or other patients [7].

In 2014, we developed and evaluated a mobile app to assist with initial allied health (AH) assessments when interpreters are not present [8]. The tool is not a replacement for interpreters but instead provides a means to support initial consultations and prompt informative patient-clinician interactions when an interpreter is unavailable. Its value is in enabling patient engagement and participation in basic interactions, expediting appropriate care, improving patient experience, and reducing costs associated with delays in care provision. It comprises key phrases and accompanying images, audio, and video content to convey key concepts between an AH clinician and a patient. The phrases were translated into 10 languages.

Senior nursing staff, who had observed the AH app in practice, expressed a significant need for a nursing-based app using the same concept. Language barriers can make it difficult for nurses to provide appropriate care to patients [9]. However, unlike AH clinicians, nursing staff are required to assist patients with daily care, including tasks as simple but as essential as providing support with eating, locating and fitting reading glasses, and assessing pain. Although nursing staff may be able to access interpreters for risk assessment and to support consent and discharge, interpreting services may not be available for assisting communication during the daily care of patients. These interactions between patients and nursing staff are more frequent and of a shorter duration than interactions between patients and other health care professionals, meaning that interpreter use is not always possible or practical. Using friends, relatives, and bilingual staff to facilitate communication with patients from NESB may at times be an option but is not an ideal alternative due to their shortage of time and lack of specific knowledge about different procedures [10].

As a response, in 2017, we worked with nursing staff to extend the original AH app by including nursing as a new module [11]. This paper reports on an evaluation of the new app with nursing staff, introduced at multiple cites of an Australian health service. This evaluation aims to quantify the value in the use of the communication app to assist nursing staff during the provision of standard care to patients from NESB when an interpreter is not available. Participants were recruited to gather use information and provide feedback to assist us in determining the impact of the use of the app and to inform any refinements required for large-scale rollout. Specifically, this project aims to determine (1) staff acceptance and satisfaction levels, (2) patient acceptance and satisfaction levels, and (3) efficacy of the app.

### Related Work

The most commonly used communication methods for patients from NESB include basic English and gestures [11]. These are speculative, time consuming, inadequate to meet all communication needs, and frustrating for both patients and nurses. As a response, mobile technology has been proposed as a potential solution to interpreter availability, with web-based tools and apps available for use. Google Translate [12], for example, is a generic tool that allows people to translate text and audio in over 90 languages. Google Translate requires internet access, which can be problematic in a hospital setting. Of greater concern, however, are the varying levels of accuracy depending on language [13], with low accuracies reported for even simple medical terminology [14]. Low translation accuracy in serious health situations will, at minimum, cause distress and, at worst, could lead to patient harm [15].

In response to this drawback, a number of purpose-built medical translation apps have been introduced to facilitate communication with patients across multiple languages, including MediBabble [16], Universal Doctor Speaker [17], Xprompt, Canopy Medical Translator [18], and BabelDr [19]. Although all these apps use text and audio to communicate, previous work from cultural advocacy groups shows that communication with CALD communities can be improved by using a variety of formats, including audio-visual and pictorial resources [20]. In addition, although current purpose-built medical translation apps include questions and phrases for clinicians to communicate with patients, there is no functionality for the patients to respond. The ability to seek accurate responses from patients is a key requirement in an environment in which accuracy is relied upon.

Furthermore, BabelDr is a novel tool developed by the Geneva University Hospitals (Hôpitaux Universitaires de Genève) in response to the refugee crisis in Europe. BabelDr is a speech-enabled, fixed-phrase translator. Similar to our approach, it relies on pretranslated sentences, but it includes speech recognition to allow doctors to search for phrases by asking questions instead of searching for them in a list. Unsurprisingly, preliminary testing showed that BabelDr is significantly more precise than Google Translate and presents higher usability than MediBabble [21]. Despite the sophisticated speech-based search, the app still requires patients to use nonverbal responses. To our knowledge, the only communication app that has been evaluated in a clinical setting is Xprompt, with participants generally supporting the introduction of mobile apps to support communication with foreign language patients, but not very enthusiastic about Xprompt’s practical use as it was perceived to be too time consuming in relation to the expected benefit [22].

### The CALD Assist App

The CALD Assist app is different from the abovementioned apps in a number of ways [8,11]. It is a communication tool specifically developed to support communication with patients from NESB when an interpreter is not available. It facilitates basic communication needs to provide appropriate care. The focus of the AH app was on patient screening. The articulated clinical need was the desire to conduct basic screening to ensure patient safety in areas such as safe swallowing, walking aides, and wound care.

Nurses unsurprisingly interact with patients in a very different manner. Nursing staff engage with patients more frequently and have information requirements around tending to the patients’ day-to-day needs. Thus, the nursing module includes additional phrases relevant specific to nursing needs but also represents a different communication challenge to the previous app.

The app’s content and functionality were gathered through user-centric design activities focused on two end user groups: clinicians and patients [8,11]. The app includes over 200 commonly used phrases professionally interpreted into 11 languages (including English) and grouped by discipline: dietetics, speech pathology, podiatry, physiotherapy, occupational therapy, and nursing. Languages were identified based on the interpretation of the historical demand of services. Each phrase is accompanied by answer options to facilitate two-way communication between the patient and the clinician.

The CALD patient user group is usually older, with varying literacy levels and potential audio and visual impairments. Thus, the app includes multimodal communication mediums, including text, imagery, audio, and video content, to increase its utility. The basic functionality includes the ability to select a language and discipline to communicate with a patient. Phrase groupings within disciplines follow the typical flow of a clinical interaction from introductions phases such as “Hello, I am your Podiatrist. I am here to talk to you about your feet,” question or assessment phrases such as “Do you have pain in your feet?,” education phrases such as “Do not get the wound wet,” and phrases to close the conversation such as “I will return with an interpreter.”

**Figure 1:**
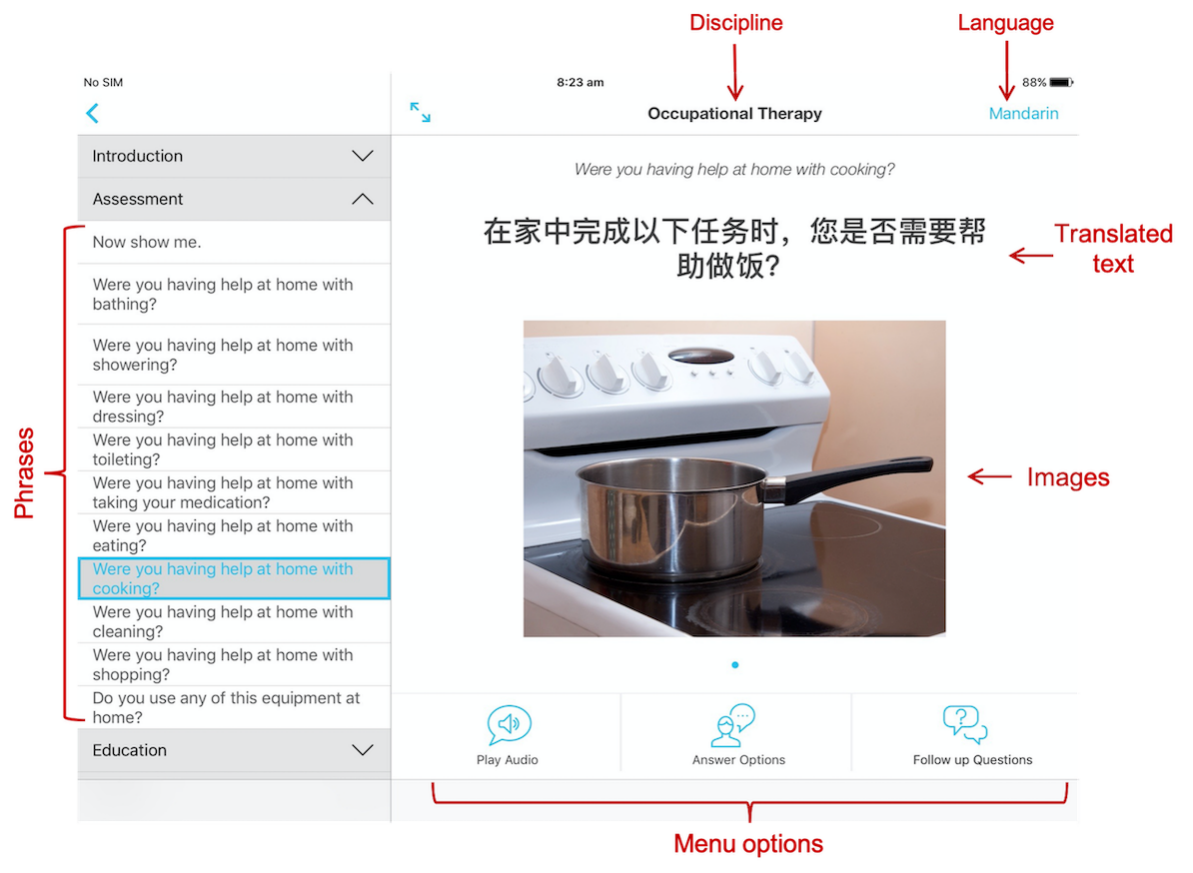
Example screenshot of the app, with Mandarin selected as the language and occupational therapy selected as the discipline.

Selection of an individual phrase reveals the phrase in the language selected in a large font, accompanied by a smaller English font for the clinician, and appropriate images or videos relating to the phrase (Figure 1). The app also allows clinicians to play prerecorded audio of the interpreted phrase and provides patients with the ability to respond to clinicians by providing *answer options* and *follow‐up questions* (that may include text and images) for many of the questions. The ability to seek detailed information from patients through two-way, multimodal communication is a key advantage over similar apps.

The AH component of the app was trialed for 6 months in a controlled introduction in an Australian health care network [8]. The free app is now available for smartphones and tablets in the Apple App and Google Play Stores.

## Methods

### Overview

**Figure 2:**
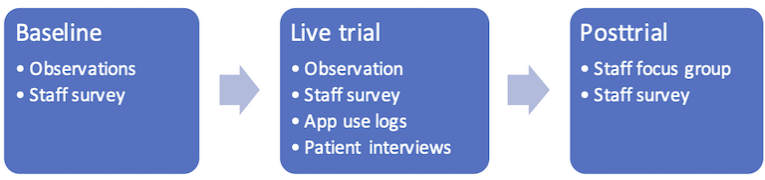
Study design divided by baseline data collection (February to June 2017), live trial (July to October 2017), and posttrial data collection (November 2017).

A study was conducted to evaluate the impact of the mobile app during the provision of care to patients from NESB. The study followed a pretest-posttest, sequential explanatory mixed methods research method. The evaluation was divided into three stages (Figure 2):

1. Baseline: The aim of this stage was to collect information regarding the standard of care and interactions in areas where the app was intended for use, including data regarding the number, mode, and length of interactions between nursing staff and patients and staff perspective on the quality of patient-staff interactions before the introduction of the app.
2. Live trial: This stage aimed to quantify the impact of the new app in terms of the number, mode, length, and quality of interactions between nursing staff and non–English-speaking patients during the provision of standard care when an interpreter was not available.
3. Posttrial: In this stage, feedback from the nursing staff who were exposed to the app was collected.

This study was undertaken at four medical, surgical, and subacute inpatient wards from three different campuses of an Australian health service. Low risk ethics approval was obtained from the Western Health (a hospital in Victoria, Australia) Low Risk Human Research Ethics Committee (LNR/16/WH/200) in January 2017. An information sheet was provided to all invited participants. Signed consent was obtained from all participants.

### Participants

Recruitment of staff participants for the baseline and live trial was through a 10-minute presentation given to nurse unit managers (NUMs) from the same participating wards during a dedicated meeting. NUMs then identified suitable staff from each ward for potential recruitment into the study. All identified staff were invited to participate via a short (2-5 minutes) presentation delivered by a member of the project team during ward meetings. A total of 99 staff members agreed to participate.

Recruitment of patient participants for interviews was conducted with the assistance of hospital interpreters. Patients who met the inclusion criteria (adults from NESB who used the app during the trial and were cognitively able to provide consent and feedback) were identified via a daily 5-minute discussion with NUMs and invited to participate in person by a member of the research team. The patient’s cognitive ability was assessed by the NUMs based on either the patient’s medical history or their own clinical judgment.

In addition, after the live trial, up to 5 staff members per ward who were exposed to the app were nominated by the NUMs as potential participants in the posttrial focus groups and invited to participate by a researcher from the project team.

### Power Calculations

Power calculations for the observations and surveys were based on the ratings of patients’ understanding of the interaction, using two independent groups of participants. With anticipated means of n_1_=3 (baseline data) and n_2_=3.5 (analysis), an SD of 0.8, α=.05, and power=80%, we anticipated a minimum sample of 41 participants per group.

The number of focus groups was guided by theoretical saturation, whereby if no new or useful information emerged, attendance ceased. The number of patient interviews was limited by the recruitment difficulties. Recruitment of patient participants was challenging, largely due to the limited number of interpreters and the patients’ inability to provide informed consent.

### Baseline Data Collection

Data regarding the number, length, and mode of interactions between nursing staff and patients were collected through observation sessions of staff-patient interactions. Observation sessions were 1 hour long, once per day, 3-5 times a week between 7 AM and 7 PM from February to June 2017. Times for each observation were randomly selected. Immediately after the hour-long observations, the staff’s perspective on the quality of patient-staff interaction was collected through paper-based surveys completed by staff participants who had interacted with a patient during the observation period. For each survey, nurses were asked to answer three questions regarding their experience of communicating with patients (Table 1).

**Table 1:** Staff surveys during the baseline and the live trials.

Survey questions	Baseline	Trial	Answer options
How successful would you rate your interactions with the patient today?	✓^a^	✓	Likert scale ranging between 1 (not successful at all) and 5 (successful)
How confident are you that the patient understood what you were saying?	✓	✓	Likert scale ranging between 1 (not confident at all) and 5 (confident)
Can you identify any phrases which would be helpful to include in the new app?	✓		Free text
Did you use the app? If not, why?		✓	(1) Unable to access, (2) time constraints, (3) did not have the phrase needed, (4) did not have the language needed, (5) not appropriate, and (6) other
How useful did you find the app when communicating with your patients?		✓	Likert scale ranging between 1 (not useful at all) and 5 (very useful)
How frustrated were you when communicating with your patient?		✓	Likert scale ranging between 1 (not frustrated at all) and 5 (very frustrated)

^a^Question present.

### Live Trial

A total of 14 iPads with the preinstalled app were made available in the participating wards. Distribution of iPads was based on the number of beds in each ward as well as additional specific requests for further iPads to facilitate app use. To ensure efficient use of the app, all staff participants received training on the use of the app a week before the start of the live trial, in person, by a member of the research team at designated nursing staff meetings.

Following the same procedure as the baseline data collection stage, data regarding the number, mode, and length of interactions between staff and patients from NESB were collected through extended observations of patient-staff interactions. Observation sessions were conducted between July and October 2017, for a period of 1 hour per day, 3-5 times a week between 7 AM and 7 PM. Times for each observation were randomly selected. Immediately after the hour-long observations, paper-based surveys were completed by staff participants who had interacted with the patient during the observation period. For each survey, nurses were asked to answer five questions regarding their interaction with the patients (Table 1). The participants were also asked for additional comments about the app.

To identify patterns of app use, app logs were automatically collected by each iPad. Here, individual use sessions were defined as any use of the app with at least a single *click*. A click is represented by a single tactile interaction with the app, such as discipline selection or language selection. Considering that the average duration of patient-nurse sessions when the app is used is approximately 205 seconds (based on the observation sessions, see the *Results* section for more information), two different use sessions were differentiated when the app was not used (there were no clicks) for at least 205 seconds.

This stage was complemented with the patients’ perspective of, and satisfaction with, the app through one-to-one interviews with patients who were exposed to the app. Interviews were standardized (the same questions in the same order) and performed by the research team with assistance from an interpreter. Patient interviews included demographic information (ie, patient age, gender, diagnosis, primary language, and self-reported level of English), communication challenges faced during current basic standard care interactions due to their NESB, and perceived confidence in their current understanding and staff’s current understanding of their needs with and without the use of the new app. All interviews were audio recorded.

### Posttrial

Staff feedback and satisfaction were collected via semistructured focus groups conducted with nursing staff who were exposed to the app during the live trial. The aim of the focus groups was to elicit information from users on the efficacy of the new app, to complement the data collected during the live trial. Aspects under discussion included the context of patient-staff interactions when the app was used, changes observed in basic standard care interactions due to the introduction of the app, and general feedback about the app. In addition, participants were asked to identify phrases and functions they found most useful; how interactions were different when the app was not available; and phrases, sections, and functions that would be useful for inclusion in the app. At least two members of the research team were present in all focus groups. The focus groups lasted approximately 60 minutes and were audio recorded.

### Data Analysis

Quantitative data analysis using inferential statistics was conducted to assess potential differences between the number, length, and quality of staff-patient interactions before and after the introduction of the app. Python 3.0 (Python Software Foundation) was used for all statistical analyses.

Qualitative data from patient interviews and posttrial focus groups were transcribed verbatim and independently reviewed by 2 researchers. The data sets were brief and clear, making them easy to interpret. An inductive approach was used to determine the coding of the data, and a semantic approach was used to analyze the data by identifying explicit words. Each researcher familiarized themselves with the data and subsequently met to discuss the codes and establish agreed themes. There were no discrepancies between the reviewers, and all themes identified by both researchers were included. The final themes were then critically reviewed and discussed by the research team, with no disagreements.

## Results

### Baseline Data Collection

A total of 85 observations and staff surveys were conducted during this stage (Table 2). In total, 3 of the non–English-speaking patients were treated by nurses fluent in the patient’s first language (Vietnamese=2 and Serbian=1) and were removed from the analysis because they do not represent the nurse-patient interactions targeted by this app. Details of the baseline data results have been reported elsewhere [11]. Here, we present only a summary of the relevant results.

**Table 2:** Language background of patients observed during the baseline and live trials.

Participants’ language background	Total participants per stage, n (%)			
	Baseline (n=82)	Trial with app used (n=30)	Trial without app (n=22)	Trial total(n=52)
Bosnian	1 (1)	0 (0)	0 (0)	0 (0)
Cantonese	0 (0)	3 (10)	1 (5)	4 (8)
Croatian	2 (2)	6 (20)	2 (9)	8 (15)
English	42 (51)	0 (0)	0 (0)	0 (0)
Greek	4 (5)	2^a^ (7)	7 (32)	9 (17)
Italian	7 (9)	4 (13)	3 (14)	7 (13)
Macedonian	6 (7)	2 (7)	1 (5)	3 (6)
Mandarin	0 (0)	1 (3)	0 (0)	1 (2)
Polish	2 (2)	0 (0)	0 (0)	0 (0)
Punjabi	0 (0)	0 (0)	2 (9)	2 (4)
Samoan	1 (1)	0 (0)	0 (0)	0 (0)
Serbian	1^b^ (1)	2 (7)	1 (5)	3 (6)
Spanish	2 (2)	1 (3)	0 (0)	1 (2)
Vietnamese	14^b^ (17)	9^b^ (30)	5^b^ (23)	14 (27)

^a^One participant removed from data analysis due to resistance to care and communication.

^b^One participant removed from data analysis because they were treated by nurses fluent in the patient’s first language.

Overall, a total of 370 interactions with a mean duration of 101 seconds (SD 141) per interaction were observed between nursing staff and patients, 164 (44.3%) of those interactions with patients from NESB, and the rest with English-speaking patients. No significant differences were observed in either the number, length, or purpose of the nurse-patient interactions between the English-speaking patients and patients from NESB. However, a significant difference was observed in both the staff’s confidence in the patient’s level of understanding (t_80_=7.49; *P*<.001) and the success of the interaction (t_80_=5.69; *P*<.001) depending on whether the patient was from an English-speaking background or NESB. That is, interactions with patients from an English-speaking background were rated by staff as more successful (mean 4.81, SD 0.45) and with higher confidence (mean 4.81, SD 0.40) of the patient’s understanding than interactions with patients from NESB (mean 3.59, SD 1.30; mean 3.14, SD 1.28). The observation sessions also revealed that although patients from English-speaking backgrounds communicate in English, patients from NESB communicate in a combination of basic English and gestures. Patients from NESB also communicate using interpreters, bilingual nurses, and family members.

### Live Trial

#### Observations and Staff Surveys

A total of 55 observations and staff surveys were conducted during the live trial (Table 2). Similar to the baseline data collection stage, 2 of the patients were treated by nurses fluent in the patient’s first language (Vietnamese) and were removed from the analysis. Data from 1 Greek patient who was resistant to care and communication were also removed.

Overall, 208 interactions with a mean duration of 135 seconds per interaction were observed between nursing staff and patients from NESB. Of these, the app was used in 71 interactions with 30 patients. The app was not used at all with the remaining 22 patients. A summary of the reasons given by staff for not using the app is presented in Table 3; note that each staff member could have mentioned more than one reason.

**Table 3:** Reasons why the app was not used by 22 patients when asked “Did you use the app? If not, why?”

Reason	Times mentioned, n
App not needed; patient knew enough English	10
Family was present to help	9
Time constraints	4
Missing phrases	3
Forgot to use it	3
Missing language	2
Patient was reluctant to engage with staff	2
My (staff’s) inability to use technology	1
Patient cognitive impairment	1

To assess the differences that may exist between the number, length, success, and quality of interactions between patients from NESB before and after the introduction of the app, a total of four one-way analyses of variance were conducted, with the length, number, confidence, and success of interactions (as reported by staff in Table 1) as the dependent variable and the following groups as the independent variables: (1) patients from NESB before the introduction of the app; (2) patients from NESB after the introduction of the app, when the app was not used; and (3) patients from NESB after the introduction of the app, when the app was used.

No significant differences were found in the number of interactions (*F*_2,89_=2.87; *P*=.06; η^2^=0.061) per observation session. Significant effects were found for the length of interactions (*F*_2,369_=11.26; *P*<.001; η^2^=0.058] as well as the confidence (*F*_2,119_=13.50; *P*<.001; η^2^=0.185) and success (*F*_2,119_=8.17; *P*<.001; η^2^=0.121) of the interactions. Post hoc comparisons using the Tukey honestly significant difference test indicated that the ratings of confidence and success of interactions with patients from NESB when the app was used were significantly higher than the ratings when the app was not used either before or after the introduction of the app. Interactions when the app was in use were also of a longer duration (Table 4).

**Table 4:** Number of samples, mean, and SD of observation variables for patients from non–English-speaking backgrounds before (during baseline) and after the introduction of the app (live trial).

Category		Before		After (with app)		After (without app)		*P* value
		Sample	Value, mean (SD)	Sample	Value, mean (SD)	Sample	Value, mean (SD)	
**Observations**								
	Number of interactions per participant	40	4.10 (2.10)	30^a^	4.67 (2.99)	22	3.09 (1.66)	.06
	Length of each interaction (seconds)	164	110.95 (148.77)	71	205.56 (185.35)	137	100.10 (156.78)	<.001
**Surveys**								
	Confidence of the interaction	40	3.24 (1.28)	30	4.23 (1.07)	52	2.77 (1.29)	<.001
	Success of the interaction	40	3.59 (1.30)	30	4.30 (0.99)	52	3.21 (1.18)	<.001
	Staff frustration	0	—^b^	30	1.5 (0.94)	52	2.23 (1.29)	.008

^a^These participants used the CALD Assist app at least once during the observation period.

^b^This question was not asked during the baseline stage.

In addition, an independent samples two-tailed *t* test was conducted to compare the reported level of staff frustration during interactions with patients from NESB when the app was used and when the app was not used. The results suggest that a significant difference exists in the reported levels of frustration (t_80_=2.71; *P*=.008; *r*=0.29), with lower frustration when the app was used (Table 4).

When asked about the usefulness of the app to nursing staff, 93% (28/30) of staff participants agreed that the app was useful for communicating with patients from NESB (very useful=18 and somewhat useful=10). When asked for additional comments about the app, staff participants suggested new languages, phrases, and images and a new feature that allowed any phrase to be typed and translated by the app. They also mentioned that although the iPad size is appropriate, the audio should be louder. They acknowledge that it takes some time to get used to the app, but they expect that the more they use it, the easier it will get. They also highlighted that to make the app more accessible, the iPads should be at the patient’s bedside or on equipment trolleys:

[I] need to get used to it. Once I’m more familiar with it, it will make it easier to use.

Nurse 24

They need to be more accessible, for example, bedside – have it already there with the patient; handover – put on the handover that they have an iPad already there to use.

Nurse 25

The observation sessions and surveys also revealed that, similar to the baseline data, patients from NESB communicate with staff using basic English, gestures, family members, and bilingual nurses (in addition to using the iPads). During these observations, the use of interpreters was not common. No significant differences in the purpose of interactions were found between the observations before and after the introduction of the app.

#### Patient Interviews

Recruitment of patient participants who used the app was more challenging than anticipated, largely due to the limited number of interpreters available and, thus, the patients’ inability to provide informed consent. A total of 7 patients (male=4 and female=3) from three language backgrounds (Vietnamese=5, Greek=1, and Croatian=1) were interviewed across three of the four trial wards. Patients were aged between 48 and 90 years. All patients identified that their level of spoken English was *a little,* with their level of English understood ranging from *a little* to *a lot*.

In total, 86% (6/7) of patient participants reported that it was not easy to communicate with their nurse, and at times, they did not understand their nurse and could not communicate their needs: “Sometimes the nurses tells me something but I didn’t understand” [Patient 2]. All patients used the app and could recall the app being used by their nurse to communicate, with 86% (6/7) indicating that it was useful, it assisted them in understanding their nurse, and it assisted their nurse in understanding their needs:

If I have an iPad it’s easier for me to communicate with them.

Patient 2

I understand and then I can answer the questions.

Patient 3

Patients mentioned additional phrases that would be helpful for inclusion, including “I need your help,” “I’m hungry,” “I’m thirsty,” “I need to go to the toilet,” and “I’m cold.” They also requested additional phrases for them to explain where the pain is and how to describe it.

#### Log Data Analysis of App Use

A total of 396 sessions were identified across all wards between July 1 and October 13, 2017. We could not distinguish between familiarity sessions and use in standard care. Sessions averaged a total of 25.6 clicks over a period of 150 seconds. The most frequently used language was Vietnamese (Table 5).

There were 1000 clicks across all categories. Of the categories selected, the most popular was pain-related phrases, which was selected 14.1% (141/1000) of the time (Table 6). We also examined the use of the individual functions of the app. Playing the audio was the most popular feature (Table 6).

**Table 5:** Number of sessions per language (N=396).

Language	Sessions, n (%)
Vietnamese	117 (29.5)
Croatian	52 (13.1)
Cantonese	48 (12.1)
Italian	39 (9.8)
Macedonian	37 (9.3)
Greek	32 (8.1)
Spanish	24 (6.1)
Serbian	22 (5.6)
Mandarin	11 (2.8)
Arabic	9 (2.3)
English	5 (1.3)

**Table 6:** Top 10 most used phrase categories and functions.

Variable		Total clicks, n (%)
**Phrase category (n=1000)**		
	Pain	141 (14.1)
	General	94 (9.4)
	Continence	74 (7.4)
	Hygiene	73 (7.3)
	Observations	69 (6.9)
	Introduction	68 (6.8)
	Mobility	65 (6.5)
	Procedures	64 (6.4)
	Use of the app	59 (5.9)
	Nutrition	55 (5.5)
**Function (n=5080)**		
	Play audio	1279 (25.2)
	Choose phrase by selecting a discipline	1073 (21.1)
	Choose category	1000 (19.7)
	Choose discipline	683 (13.4)
	Choose language	328 (6.5)
	Swipe image	199 (3.9)
	Phrase image selected	193 (3.8)
	Show answer options	141 (2.8)
	Choose phrase by browsing or searching	105 (2.1)
	Searching started	76 (1.5)

### Posttrial Focus Groups

#### Overview

Three focus groups were conducted at the same three campuses of the Australian health service. The focus groups brought together 1 NUM and 8 nurses from the participating wards, including: (1) respiratory and infectious disease; (2) upper gastro-intestinal surgery; (3) ear, nose, and throat surgery; (4) plastics and thoracic surgery; (5) geriatric evaluation and management and rehabilitation; and (6) oncology, gastroenterology, hematology, renal, and endocrinology. Qualitative analysis focused on four practical themes of app deployment: app use, context of use, content and functionality, and accessibility.

#### Nurses’ Description of App Use

Participants believed that the CALD Assist app facilitated basic communication needs with patients from NESB, and helped them deliver the care the patients needed. They mentioned that with the app, there was less need to seek assistance from family members or bilingual colleagues. They reported that they would use the app as the first resource but go to interpreters or family members if they needed additional help.

There were no reports of resistance to using the app by either patients or family members. Nurses believed that patients felt more included using the app, and family members appreciated it. It was highlighted, however, that patients with cognitive impairment had difficulty understanding long sentences. One participant reported *embarrassment* due to her inability to find the phrases she needed in front of a patient. This sense of embarrassment prevented her from using the app more often:

...you will be using your iPad in front of the patient and trying to find it and then is embarrassing that you couldn’t find the one that is suitable to communicate...[then] I didn’t really used it for a while.

Nurse 1

Participants also highlighted two stories that demonstrated the impact of the app. In one story, they described how the app was used to facilitate a pain medication dosage change in a patient, whereas in the other story, the app was used to help nurses find out that a patient had chest pain. According to participants, without the app, these exchanges would have been more difficult:

...we wanted to know if the pain lessened or higher [sic]. They [doctors] use the iPad because they want to change that [the dosage].

Nurse 2

We had a lady who was...saying that she had pain and I was trying to determine if it was chest pain. We used the pain questions even though it’s not “Do you have chest pain or abdominal pain?” while pointing.

Nurse 3

#### Context of Use

It was mentioned that the app was particularly useful for older patients from NESB who tended to speak and understand less English than younger generations. Participants also believed that the app could be useful in communicating with patients with hearing impairment, as long as they were able to read. Participants highlighted that the app allowed them to do more for patients because communication was easier, quicker, and more accurate:

…you’re more sure of what she wants and it’s quicker...because you don’t have to guess?

Nurse 4

#### Content and Functionality

Participants were very positive about the phrases, sections, and functionality of the app. According to them, the app is easy to use, the text is large enough for patients to read, and the pictures are useful and appropriate. The audio is good but can be louder. The spoken phrases are appropriate for most patients but too fast for some of them, and participants suggested a function to slow down the audio when needed. Participants also suggested additional functionalities such as voice activated functions, the ability to translate spoken text into or from the patient’s language (similar to Google Translate), and a calendar and time function to tell patients the time or date of a procedure.

Most participants noted that the more the app was used, the more comfortable they felt using it. They mentioned that the browsing option to go through phrases was used more than the search functionality. Participants who used the search function, however, believed that it was a better and faster way to find phrases: “...the more you use it, the more you know exactly what’s on there” [Nurse 7].

#### Accessibility

In two of the wards, the iPads were stored at either the nursing stations or the drug room. As a result, nurses often forgot to use them or had no time to go and get them from those locations. In the rest of the wards, the iPads were placed next to the bed of a patient that may need the CALD Assist app to communicate:

...because out of sight is out of mind for a lot of things. It’s there, make use of it, and just to encourage them [my staff].

Nurse 5

Although there were concerns that the iPads could have been damaged or stolen (none were during the trial), this approach improved access and visibility.

## Discussion

### Overview

Effective communication in clinical settings is essential. The inability of hospital staff to communicate effectively with patients from NESB can impact patient care and experience. This paper reports the results of an impact evaluation of the CALD Assist app in a controlled rollout on medical, surgical, and subacute inpatient wards at three different campuses of an Australian health service. This evaluation aimed to quantify the value of the app during the provision of standard care to patients from NESB when an interpreter is not available.

This is the first study to investigate the value of using mobile health tools to support communication with patients from NESB in clinical settings. Previous studies have focused only on accuracy and technology acceptance. Thus, a key contribution is the demonstration that a multimodal app can affectively assist communication between nurses and patients from NESB in the absence of interpreters; reducing care inequities between patients from English-speaking backgrounds and NESBs, increasing staff confidence, and reducing staff frustration.

### Principal Findings

Baseline observations and surveys confirmed that significant differences were observed in nurse-patient interactions depending on whether the patient identified as English speaking or from a NESB. Nurses treating patients from NESB scored lower in both their perception of the patient’s level of understanding and the success of the patient-nurse interaction. This reinforced the need for a tool to support communication access with patients from NESB. Interestingly, no significant differences were observed in the type, length, or number of interactions between staff and English-speaking patients or patients from NESB.

The observed interactions and the postobservation surveys also confirm that the main purpose of interactions was consistent with those reported during the design and development stages of the app [11], including pain management, mobility, hygiene, and nutrition.

During the live trial, the app was used by nursing staff in 396 sessions to provide standard care to patients. Although we do not know the exact number of staff-patient interactions during the same period, during the observations, from a total of 208 interactions with 58% (30/52) of the patients, the app was used in 71 (34.1%) interactions. Given that the app was new and only available on four wards, the number of sessions conducted was encouraging. It is expected that the app will be used more regularly, as nurses become more familiar with it.

In addition, 93% (28/30) of staff participants believed that the app was useful to communicate with patients from NESB when an interpreter was not present, and 86% (6/7) of patients indicated that the app assisted them in communicating with their nurse. Through the use of the app, a number of patients from NESB experienced better provision of standard care, similar to their English-speaking peers. That is, interactions with patients from NESB were rated as more successful, and staff report of *confidence* in patients’ understanding increased (to levels similar to their English-speaking peers) when the app was used, independent of the availability of the app. In addition to the app, participants communicate with patients using gestures, family members, and interpreters.

Speaking a second language is also a real benefit for staff, as they can communicate with patients more easily than by using any of the other approaches. Although bilingual nurses were excluded from the study, it was noted that the three interactions recorded between patients from NESB and staff members fluent in the patient’s first language were rated as the highest score on the 5-point Likert scale in all cases for the level of understanding and success of the interactions. Although the data are limited, this result supports previous findings that suggest that bilingual staff can assist in improving the quality of care for patients from NESB [9].

Staff and patients were all positive about the app and its content and functionality. We note the high utilization of pain, general, continence, and hygiene phrases that allow nurses to provide basic care to patients. This distribution of categories selected was expected, given the priorities mentioned by staff members during the user needs stage of the project [11]. In fact, the creation of phrase groupings aimed to facilitate flow in typical scenarios of use. It is possible that using these groupings might have facilitated the interaction between participants and, therefore, influenced the app evaluation. We also note high use of the language, phrase, and category selection and low use of the search capability. We expect that through increased familiarity, we will see increased use of this function.

### Challenges and Limitations

The introduction of the app was not without any challenges. The introduction of new clinician‐focused technology in a hospital environment is complex, as experienced clinicians find it challenging to change established behaviors or practices. As a result, they may follow current practices without considering the new app as a tool to facilitate standard practice. Training and familiarity with the app and the phrases played a significant role in the participants’ use and experience. Following our evaluation, an education and promotion stage was introduced to further embed the use of the new app into current practice.

Finding the ideal location that provided visibility and accessibility to the iPad, while ensuring the security of the device, was essential. Positioning the iPads close to the patient’s bed proved to be ideal.

Although the app was reported to be effective when used to assist patients with mild cognitive impairment, it was not specifically designed for this population. In this trial, cognitive impairment was reported by participating nurses in 6 participants, and the app was reported to be useful in 4 (67%) of those cases.

Finally, although the impact of the app on staff-patient interactions was evaluated via observations and a short survey, validated scales to measure the usability and acceptability of the app were not used (eg, the system usability scale or the technology acceptance model) to reduce the participation load on nursing staff who lack time. Future research should further evaluate the usability and acceptability of the app.

### App Refinements

Our results suggest that the app was used particularly by older patients. The current version of the app, however, appears to be limited for patients with cognitive impairment, and additional material based on keywords and short phrases (eg, “Toilet?” with answer options yes or no) is recommended for this population.

Although both male and female voices were suggested during the design stage of the app to address potential cultural and life experience concerns (eg, female patients with a history of sexual assault might be uncomfortable with a male voice) [11], the current version includes only a single voice per language.

### Conclusions

This study guides the impact evaluation of a communication app to directly improve the provision of care to patients from NESB. Using the proposed app, nursing staff delivered safer, higher quality care to a potentially at-risk and vulnerable population, reducing inequity in health care delivery and providing a timelier and more positive patient experience to patients.

## Abbreviations

AH: allied health
CALD: culturally and linguistically diverse
NESB: non–English-speaking background
NUM: nurse unit manager
